# Dataset of certified food dye levels in over the counter medicines and vitamins intended for consumption by children and pregnant women

**DOI:** 10.1016/j.dib.2020.106073

**Published:** 2020-07-31

**Authors:** Arlie L. Lehmkuhler, Mark D. Miller, Asa Bradman, Rosemary Castorina, Alyson E. Mitchell

**Affiliations:** a Department of Food Science & Technology, University of California-Davis, 1 Shields Ave, Davis, CA 95616, USA; b Office of Environmental Health Hazard Assessment, California Environmental Protection Agency, Sacramento, CA, USA; c Center for Environmental Research and Children's Health (CERCH), School of Public Health, University of California, Berkeley, CA, USA

**Keywords:** FD&C dyes, Vitamins, Over the counter medications, High performance liquid chromatography

## Abstract

Food, Drug, & Cosmetic (FD&C) dyes can be found in various products outside of food that are consumed by children. The amount of FD&C dyes used in commercial products is proprietary. Determining the contribution of dye intake from commercial products requires direct assessment of FD&C dyes in the products. This dataset contains the raw data of HPLC peak areas, absolute values, averages, SDs and % RSD for FD&C dyes in children's gummy vitamins, children's tablet vitamins, prenatal vitamins, children's cough/cold/allergy tablets & syrups, and children's pain reliever tablets & syrups obtained using high performance liquid chromatography with a photometric diode array detector (HPLC-PDA). The data can be used for further interpretations of dye intake in children, based upon dose levels suggested for distinct age groups, to evaluate the consumption of the FD&C dyes and accepted daily intake (ADIs) suggested for each FD&C dye by the United States Food & Drug Administration (US FDA). The variability associated within each category is critical for understanding how products on the market can differ between lot especially with large gaps between expiration dates. The interpretation of the data is described in “Certified Food Dyes in Over the Counter Medicines and Supplements Marketed for Children and Pregnant Women” in the Journal of Food and Chemical Toxicology [1].

**Specifications Table**

**Table:** 

**Subject**	Analytical Chemistry
**Specific subject area**	Toxicology, Public Health
**Type of data**	Tables of raw data showing peak area from HPLC, adjustment for internal standard, associated concentration on calibration curve, and final calculation for each sample. Tables of absolute values, averages, SDs and % RSD of Food, Drug, & Cosmetic (FD&C) dyes in children vitamins and over-the-counter medications (OTCs) marketed for children and pregnant women.
How data were acquired	Dyes were obtained using an InfinityLab Poroshell 120 EC-C_18_ column (250 mm × 4.6 mm, i.d. with 4 μm particle diameter, Agilent Technologies, Memphis, TN) on an Agilent 1200 HPLC system coupled with a photodiode array (PDA) detector (Agilent Technologies, Memphis, TN). Software: ChemStation Rev. B.04.03
**Data Format**	Raw Data
**Parameters for data collection**	Three to five brands of children's gummy vitamins, children's tablet vitamins, prenatal vitamins, children's cough/cold/allergy tablets & syrups, and children's pain reliever tablets & syrups were collected across three U.S. states (California, Indiana, and Georgia) were sampled in varying flavors/colors, if possible. Three different lots, with differing expiration dates, of each brand were selected for sample replication. Each lot was extracted in duplicate.
**Description of data collection**	A composite sample was created for each lot of each brand and the sample was diluted in water, then sonicated until homogenous. The sample was centrifuged before being concentrated and washed on a solid phase extraction (SPE) column. The final sample collected was dried in a Speed Vac and reconstituted in water to be quantified using HPLC-PDA.
**Data source location**	University of California-Davis, Davis, California
**Data accessibility**	Repository Name: Mendeley Data Data identification number: 10.17632/n6m7rhct2h.1 Direct URL to data: http://dx.doi.org/10.17632/n6m7rhct2h.1
Related research article	Lehmkuhler, M. Miller, A. Bradman, R. Castroina, A. Mitchell, Quantification of Certified Food Dyes in Over the Counter Medicines and Supplements Marketed for Children and Pregnant Women. In press.

**Value of the Data**

• Those working in public health and allied fields can benefit from this data to understand that variable can be dependent on lot differences (processing), brand, and product expiration date due to dye degradation.
• These data can further be applied to average heights, average weights, and dosing to determine the contribution of OTCs and vitamins to certified food dye intake in children.
• This data can be utilized in further investigations of dye degradation in correlation with expiration date in children's vitamin gummies. The toxicity of degradation products of FD&C dyes in these products has not been investigated.

## 1 Data description

FD&C dyes were evaluated in seven common commercial over-the-counter medicines (OTCs) and vitamins including: children's gummy vitamins, children's tablet vitamins, prenatal vitamins, children's cough/cold/allergy tablets & syrups, and children's pain reliever tablets & syrups and the seven data files are individually organized in the same manner. The FD&C dyes measured in the products include FD&C Yellow 5, Yellow 6, Red 40, Blue 2, and Blue 1. For each file, the average dye content in milligrams per kilogram can found in Table 1 plus/minus the standard deviation for each value. Table 2 shows the average mass of each tablet/gummy or average density for the syrup categories, along with the conversion to kilogram, or kilogram per milliliter when appropriate. The summary of average dye content per tablet, gummy, or milliliter can be found in Table 3 with its corresponding standard deviation and % RSD (to evaluate variability within the brand). Tables 4–8 are individual values of each sample evaluated. Tables 4–8 contain the average value of dye for each lot along with the average, standard deviation, and % RSD of dye for each brand. Below the data aforementioned in Tables 4–8, all peak areas, adjusted peak areas with internal standards, concentration based on the calibration curve, and the value after back calculating the concentration with consideration of the sample preparation process are listed. Children's gummy vitamins and children's tablet vitamins are organized by each brand having an individual table in Tables 4–8. Each individual table contains data organized by color, since these two categories had three colors within each lot for each brand. Tables 4–8 associate the expiration date with each corresponding lot for every category.

## 2 Experimental design, materials and methods

### 2.1 Materials for analysis

Analytical standards for each of the following food dyes: FD&C Yellow No. 5, Yellow No. 6, Red No. 40, Blue No. 2, Blue No. 1, and Amaranth (formerly FD&C Red No. 2) were purchased from Millipore Sigma (Missouri, USA). HPLC Grade ammonium acetate was purchased from Spectrum Chemicals (California, USA). Formic acid (High Purity Grade) and methanol (HPLC Grade) were purchased from VWR. Acetonitrile, HPLC Grade, >99.9% and hydroxide solution, ACS Reagent 28.0-30.0% was purchased from Millipore Sigma (Missouri, USA). Waters Oasis WAX 3 cc Vac Cartridges (60 mg sorbent per cartridge, 60 μm Particle Size) were used for sample preparation and obtained from Neta Scientific (New Jersey, USA).

### 2.2 Samples for analysis

Seven categories were identified to sample the OTC and vitamin market, including children's gummy vitamins, children's tablet vitamins, prenatal vitamins, children's cough/cold/allergy tablets & syrups, and children's pain reliever tablets & syrups. Each category was identified on the market to determined number of brands that could be sampled. Three to five brands were identified for each category and products with multiple colors were evaluated based on each color rather than a composite of all colors. Three lots were identified to better evaluate each brand and potential variability over different preparations of the same product. Each lot, and color if necessary, was prepared and evaluated in duplicate.

### 2.3 Standards

A mixture of each FD&C dye purchased including the internal standard (Amaranth) established an eight point calibration curve at concentrations of 0.25, 0.5, 1, 2.5, 5, 10, 25, 50 mg L^−1^ for quantification in MilliQ water.

### 2.4 Sample Preparation

Sample preparation was completed based on Yang et al (2014) and Yang et al (2011) [2,3]. Syrups and gummies were weighed out at 3 grams per sample into a 50mL conical tube. The vitamin tablets were crushed using mortar and pestle and only 1 gram was used for evaluation. The syrups and gummies were brought up to 30mL total volume using MilliQ water. The vitamin tablets were brought up to 10mL total volume using MilliQ water. Sonication and heat was used to homogenize the samples for 30 min at 55 ± 5°C; each sample was vortexed every 10 min to ensure homogeneity. Samples must be completely dissolved in order for sonication to be complete. These samples were centrifuged at 5000RPM for 10 min and set aside. An SPE manifold was used with Oasis WAX SPE Cartridges (3cc, 60 mg x 60 μm) to clean up samples. The cartridges were conditioned with 1mL of methanol and followed by 1mL of water. Three mL of each sample was loaded onto the column and pulled through the cartridge by gravity. The cartridge was washed with 1mL of 2% formic acid in water and 1mL of methanol to follow. The samples were collected in glass tubes with 2 mL of 5% ammonia in methanol solution. The glass tubes were dried using a SpeedVac (Savant SpeedVac, ThermoFisher, Massachusetts, USA). The internal standard was added after drying (10µL of 250µg/mL amaranth in water) and brought up in 500µL of MilliQ water. The sample was put into a vial for HPLC analysis.

Tablet samples from children's cough/cold/allergy and children's pain reliever categories containing FD&C Blue No. 2 were evaluated by measuring the crushed composite out to 0.1g sample and added to a 2mL centrifuge tube with 0.01g EDTA. Sodium hydroxide solution at 0.25M was added for a final volume of 2mL. The samples were vortexed and sonicated for 30 min at 40 ± 5°C. Each tube was centrifuged for 10 min at 12000RPM and the supernatant was transferred to a new 2mL tube to adjust the pH back to 6-7 with 5% formic acid solution in water. The SPE procedure described above is the continuation of sample preparation for these samples, only using 1mL of sample rather than 3mL as described for syrups, gummies, and other tablets.

### 2.5 Instrument conditions

HPLC analysis was completed on an Agilent 1200 HPLC system coupled with a photodiode array (PDA) detector (Agilent Technologies, Memphis, TN) using an Agilent InfinityLab Poroshell 120 EC-C_18_ column (250 mm × 4.6 mm, i.d. with 4 μm particle diameter, Agilent Technologies, Memphis, TN). A mobile phase composed of A (10 mmol L^−1^ ammonium acetate in water) and B (acetonitrile) was used to achieve peak resolution. The gradient elution of 0 min (3% B), 0−2 min (3% B), 2−5 min (10% B), 5−10 min (30% B), 10-12 min (33% B), 12−15 min (3% B), 15-17 min (3% B) at a flow rate of 1 mL min^−1^ was used for highest efficiency and peak resolution. The column temperature was maintained at 25°C with a 50µL injection volume. Peak detection occurred at 420 nm, 480 nm, 520 nm, 609 nm, and 620 nm with the full scan PDA ran over 200–650 nm. Peak identification was confirmed by retention time and spectral characteristics of the standards.

## Declaration of Competing Interest

The authors declare that they have no known competing financial interests or personal relationships which have, or could be perceived to have, influenced the work reported in this article.

## Appendix Supplementary materials

Image, application 1
